# Replication stress increases mitochondrial metabolism and mitophagy in FANCD2 deficient fetal liver hematopoietic stem cells

**DOI:** 10.3389/fonc.2023.1108430

**Published:** 2023-03-07

**Authors:** Makiko Mochizuki-Kashio, Noriko Otsuki, Kota Fujiki, Sherif Abdelhamd, Peter Kurre, Markus Grompe, Atsushi Iwama, Kayoko Saito, Ayako Nakamura-Ishizu

**Affiliations:** ^1^ Department of Mieroscopic and Developmental Anatomy, Tokyo Women’s Medical University, Tokyo, Japan; ^2^ Institute of Medical Genetics, Tokyo Women’s Medical University, Tokyo, Japan; ^3^ Department of Hygiene and Fublic Health, Tokyo Women’s Medical University, Tokyo, Japan; ^4^ Department of Pathology, St. Jude Children’s Research Hospital, Memphis, TN, United States; ^5^ Children’s Hospital of Philadelphia, Comprehensive Bone Marrow Failure Center, Perelman School of Medicine, University of Pennsylvania, Philadelphia, PA, United States; ^6^ Papé Family Pediatric Research Institute, Oregon Stem Cell Center, Oregon Health & Science University, Portland, OR, United States; ^7^ Division of Stem Cell and Molecular Medicine, Center for Stem Cell Biology and Regenerative Medicine, The Institute of Medical Science, The University of Tokyo, Tokyo, Japan

**Keywords:** Hematopoietic stem cell, FANCD2, replication stress, mitochondria metabolism, mitophagy, fetal liver

## Abstract

Fanconi Anemia (FA) is an inherited bone marrow (BM) failure disorder commonly diagnosed during school age. However, in murine models, disrupted function of FA genes leads to a much earlier decline in fetal liver hematopoietic stem cell (FL HSC) number that is associated with increased replication stress (RS). Recent reports have shown mitochondrial metabolism and clearance are essential for long-term BM HSC function. Intriguingly, impaired mitophagy has been reported in FA cells. We hypothesized that RS in FL HSC impacts mitochondrial metabolism to investigate fetal FA pathophysiology. Results show that experimentally induced RS in adult murine BM HSCs evoked a significant increase in mitochondrial metabolism and mitophagy. Reflecting the physiological RS during development in FA, increase mitochondria metabolism and mitophagy were observed in FANCD2-deficient FL HSCs, whereas BM HSCs from adult FANCD2-deficient mice exhibited a significant decrease in mitophagy. These data suggest that RS activates mitochondrial metabolism and mitophagy in HSC.

## Introduction

FA is an inherited BM failure with hematologic disease onset around school age (1). Yet, murine models and studies on FA patients have revealed that genetic mutation of FA genes affects embryonic HSC function (2–4). FA HSCs exhibit a decrease in number in the fetal liver where HSCs should rapidly expand. Compared to predominantly quiescent adult BM HSCs, FL HSCs are highly proliferative around E13.5 and show a strong RS response. We previously showed that FA FL HSCs exhibited significantly greater RS compared to wild-type (WT) FL HSCs due to delayed recovery from replication fork collapse (5).

Recently, we and others have highlighted mitochondrial metabolism as a critical component for maintenance in adult quiescent HSCs (6, 7). Mitochondrial membrane potential (MMP) is lower in HSCs than in progenitor cells but HSCs with large mitochondrial mass exhibit high stem cell potential (8). Due to their proliferation, FL HSCs exhibit higher mitochondrial activity compared to adult HSCs (9). Mitochondria oxidative phosphorylation and TCA cycle-associated gene expression are also higher in FL HSCs compared to adult BM HSCs. Others previously reported that mitochondrial metabolism and mitochondria clearance by mitophagy was reduced in FA cells (10–12). Moreover, HSC number in FA mice were rescued by treatment with the mitochondrial complex I inhibitor, metformin (13).

To understand how mitochondrial metabolism affects FA pathophysiology, we hypothesized that RS during FL HSC expansion modulates mitochondrial metabolism. Using pharmacologically induced RS, we observed temporary alterations in MMP and increased mitophagy. We then analyzed FL and BM HSCs from Fancd2 knockout (Fancd2 KO) mice. Strikingly, MMP and mitophagy were elevated in FANCD2-deficient FL HSCs. In contrast, mitophagy was downregulated in adult BM HSC. Taken together these observations indicate that the pathophysiological RS in FANCD2-deficient fetal HSCs boosts mitochondrial metabolism and mitophagy.

## Material and methods

### Animal husbandry

C57/BL6 background Fancd2 KO mice (14) were kindly gifted by Dr. Markus Grompe (OHSU). mitoDendra mice were obtained from JAX (#018397). All animal experiments were approved by TWMU animal experiment committee.

### Flowcytometry (FACS) analysis

HSC immunophenotyping analysis was conducted using antibodies for EPCR, CD150, CD48 (HM48-1), c-Kit (2B8), Sca-1 (D7, excluded when analyzing 5-FU treated mice) and Lineage (Gr-1 (RB6-8C5), Ter119 (Ter-119), B220 (RA3-6B2), CD4 (RM4-5), CD8 (53-6.72), Mac-1 (M/70) excluded when analyzing FL HSCs). MMP was measured with tetramethylrhodamine (TMRE) 200nM and used 488nm lasor/585-642nm detector. mtROS was measured with MitoSoxRed (M36008, Thermo Fisher) and used 488nm lasor/585-642nm detector. Lysosome acidification was detected with Lysotracker Red (LTR, L7528, Thermo Fisher) and used 638nm lasor/660-720nm detector. Mitophagy activity was detected with Mitophagy detection kit (mtPH, MD1, Dojindo) and used 488nm lasor/780-860nm detector.

To perform intracellular staining, cells were fixed and penetrated using IntraPrep (A07803 Beckman) and stained with anti-p4EBP (T37/46, CST2855) 1:200.

All data were measured by CytoFlex FACS analyzer (Beckman Coulter). Analysis is performed with FlowJo software.

### O-propargyl-puromycin (OPP) protein synthesis assay

Newly synthesized protein was measured. E13.5 FL cells or Mom’s BM were cultured with 10mM O-propargyl-puromycin (OPP) in IMDM 10%FBS for 40min, harvested and staind antibodies of HSC (CD150-PE-Cy7, CD48-AF700, c-Kit-BV421, Sca1-PE-Cy7, Lineages with PerCpCy5.5). After washed, cells are fixed and penetrated using IntraPrep (A07803 Beckman). By using Click-iT Plus OPP Alexa Fluor 488 (AF488) Protein Synthesis Assay Kit (C10456, Thermo Fisher), stained OPP with AF488 and subsequent to FACS to measure the intensity (used 488nm lasor/525-540nm detector).

### Immunofluorescence and imaging flow cytometer (IFM) analysis

HSC (CD150+ CD48- Lin- Sca-1+ c-Kit+) or HSPCs (Lin- Sca-1+ c-Kit+) were sorted by FACS AriaII or III (BD) and fixed with 4%PFA. Cells were permeabilized with 0.5% Triton for 15min and stained with anti-Tomm20 (ab78547 or ab289670) 1:200 or anti-ssDNA (18731, IBL) 1:50. Secondary stains were performed with anti-rabbit-AF488 1:1000 or anti-rat-AF488 1:1000. DAPI 1:1000 or 7-AAD 1:200 were used for nuclear staining. Data correction was performed with Mark-II (Amnis) and analysis was performed with IDEAs software (Amnis).

### 5-FU treatment

Mice were intraperitoneally injected 5-FU with one shot (200mg/kg body weight).

### *Ex vivo* HSC culture

HSCs were sorted by FACS Aria II or III (BD) and subjected to ex vivo culture. 2000 HSCs per 1 well of 96-well dish are cultured with SF-03 medium (Sekisui) supplemented with 100ng/ml SCF (455-MC, R&D), 100ng/ml TPO (488-TO, R&D) and 0.1% BSA. Aphidicolin was diluted with DMSO for 50mg/ml and used as 1:5000-10000(final concentration 50-100ng/ml).

### CFU assay

HSCs were sorted by FACS Aria II or III (BD) and 500 cells are seeded onto 3ml of mouse methylcellulose media (R&D Systems, HSC007) with APH (50-100ng/ml) or imTOR of Rapamycin (Thermo PHZ1235, diluted with DMSO for 10mM and used as 1:1000, final concentration 10μM) or iTgf-β of SD-208 (CAYMAN 16619, diluted with DMSO for 10mM and used as 1:1000, final concentration 10μM). The media was equally divided into three 3.5cm dishes and incubated at 37°C. Colony numbers were counted after 14 days.

### Statistics

Statistical analyses were performed with t-test and one-way ANOVA (if the parameter is over 2) by using prism software (GraphPad).

## Results

### RS temporarily increases HSC mitochondrial activity and mitophagy *in vivo*

**Figure 1:**
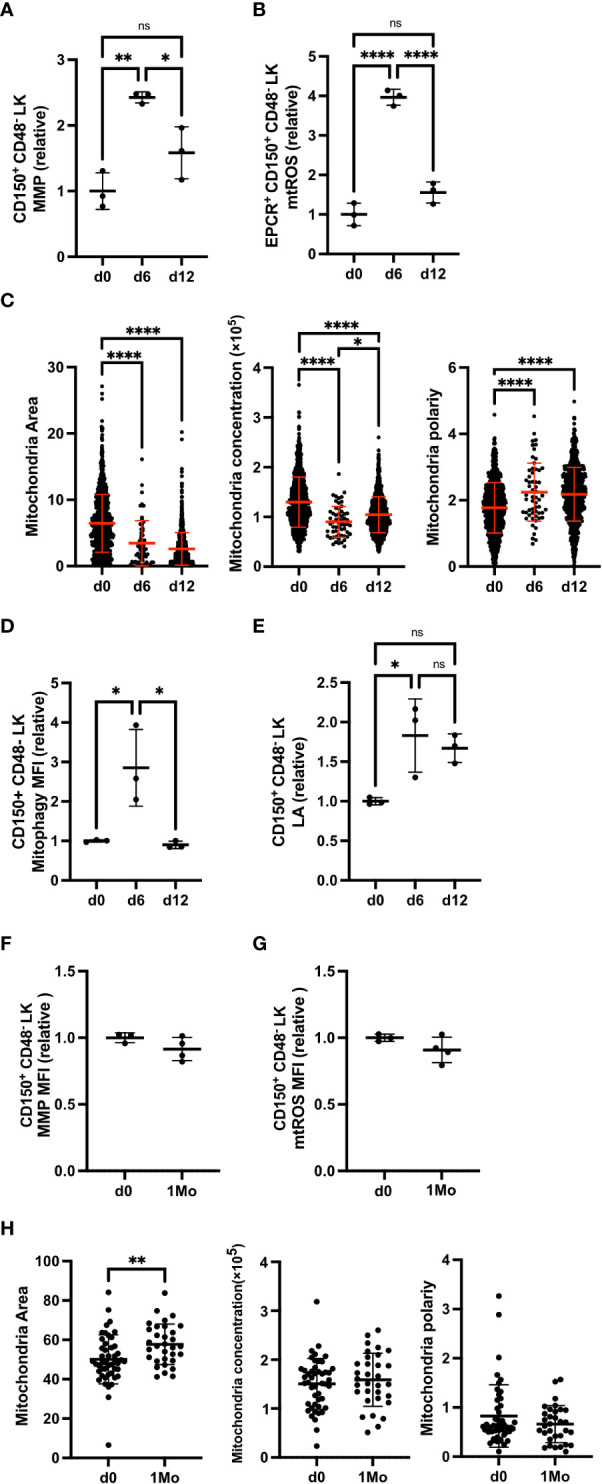
5-FU-induced RS increases MMP and mitophagy. **(A, B)** Relative mean fluorescence intensity (MFI) of MMP (TMRE) **(A)** and mtROS (MSR) **(B)** in HSPCs (EPCR+ CD150+ CD48- c-Kit+ Lin-) at untreated (d0), day 6 (d6) and day 12 (d12) after 5-FU injection (d0 n=3 mice, d6 n=3 mice, d12 n=3 mice). **(C)** Mitochondrial area, mitochondrial concentration, and mitochondrial polarity of HSPCs from mitoDendra mouse, at d0, d6 and d12 after 5-FU injection (d0 n=931 cells, d6 n=58 cells, d12 n=867 cells). Data are analyzed by IFM. **(D, E)** Relative MFI of mitophagy **(D)** and lysosome acidification (LA, LTR) **(E)** in HSPCs at d0, d6 and d12 after 5-FU injection (d0 n=3 mice, d6 n=3 mice, d12 n=3 mice). **(F, G)** Relative MFI of MMP **(F)** and mtROS **(G)** in HSPCs at 1 month (1Mo) after 5-FU injection (d0 n=3 mice, d6 n=3 mice, d12 n=3 mice). **(H)** Mitochondrial area, mitochondrial concentration and mitochondrial polarity in HSPCs at 1 month (1Mo) after 5-FU injection (d0 n=47 cells, 1Mo n=32 cells). Data are analized by IFM. All relative data are divided with average value of d0 and show mean ± SD. *P<0.05, **P<0.01, ****P<0.0001, ns, not significant.

We previously described that FL HSCs experience RS due to their proliferative nature. We also showed that RS responses are increased in Fancd2 KO FL HSCs compared to BM HSCs from adult Fancd2 KO mice (**Figure S3A**) (5). Here we tested whether RS alters HSC mitochondrial activity and mitophagy. Administration of 5 fluorouracil (5-FU) to WT mice depletes lineage cells and causes subsequent proliferation and RS in adult BM HSCs (15). It was previously described that 5-FU treatment changes Sca-1 expression so that we excluded Sca-1 marker and measured as CD150^+^ CD48- LK Hematpoietic Stem and Progenitor Cells (HSPCs) (15). After 6 days of 5-FU administration, HSPCs exhibited a significant increase in MMP (**Figures 1A**, **S1A–C**), which returned to baseline by day 12 when cells no longer proliferate. In parallel to changes in MMP, mitochondrial reactive oxygen species (mtROS) in HSPCs were also elevated at day 6 and returned to baseline by day 12 (**Figure 1B**).

We next analyzed the mitochondrial morphology in HSPCs. Alterations in mitochondrial morphology and distribution in HSCs have been previously reported in myelodysplastic syndrome (MDS) and leukemia; mitochondria in HSCs from MDS models diffuse and spread out while mitochondria in leukemic stem cells (LSC) exhibit polarity and are concentrated (16, 17). Imaging flow cytometer (IFM) was utilized to calculate mitochondrial area, concentration, and polarity in individual HSPCs. Following the 5-FU challenge, the mitochondrial area decreased and diffused, producing a polar redistribution pattern on day 6 that continued through day 12 (**Figure 1C**).

Adequate levels of mitophagy and lysosomal activity help maintain HSPCs potential (18–20). When we evaluated mitophagy and lysosomal activity, we found both increased on day 6, and HSPCs lysosomal activity remained high on day 12 (**Figures 1D, E**). Furthermore, the long-term effect of experimental RS on HSPCs was analyzed. HSPCs from mice 1 month after 5-FU injection (1Mo HSC) continued to exhibit a significantly larger mitochondrial area (**Figure 1H**), but comparable MMP and mtROS compared to control HSPCs (**Figures 1F, G**). Together, these data indicate that *in vivo* RS drastically alters MMP, mtROS, mitophagy and mitochondrial distribution in adult HSPCs.

### RS increases mitochondrial activity and mitophagy in cultured HSC

**Figure 2:**
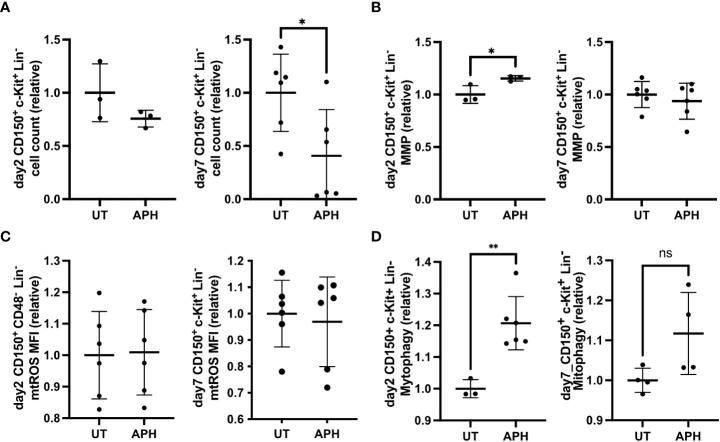
APH-induced RS increases MMP and mitophagy. **(A-D)** Relative cell count **(A)**, MFI of MMP **(B)**, MFI of mtROS **(C)** and MFI of mitophagy **(D)** of cultured HSCs (CD150+ c-Kit+ Lin-) at day 2 (left panel) and day 7 (right panel), with or without APH (UT) (day 2 UT n=3-6 wells, APH n=3-6 wells, day 7 UT n=6 wells, APH n=6 wells). All relative data are divided with average of UT and show mean ± SD. *P<0.05, **P<0.01, ns, not significant.

We next investigated changes in mitochondrial activity in cultured HSCs subjected to aphidicolin (APH) which induces RS by inhibiting DNA polymerase (21). After 7 days of culture APH-treated HSCs exhibited a significant decline in number compared to control HSCs (**Figure 2A**). APH-treated HSCs exhibited significantly fewer cell divisions compared to control HSCs (**Figure S1D**). APH-treated HSCs did not exhibit an increase in apoptosis as shown by Annexin-V staining (**Figure S1E**). Hematopoietic progenitor colony formation of HSCs was significantly increased with APH-treatment (**Figure S1F**). MMP of APH-treated HSCs transiently increased on day 2 but normalized by day 7 (**Figure 2B**). Both on day 2 and on day 7, APH-treated HSCs exhibited unaltered mtROS level (**Figure 2C**). Mitophagy activity was also significantly upregulated in APH-treated HSCs at day 2 but comparable at day 7 (**Figure 2D**). These data indicate that BM HSCs under experimental RS in an ex vivo culture upregulate MMP and mitophagy.

### *Fancd2* KO BM HSC exhibits decreased mitophagy

**Figure 3:**
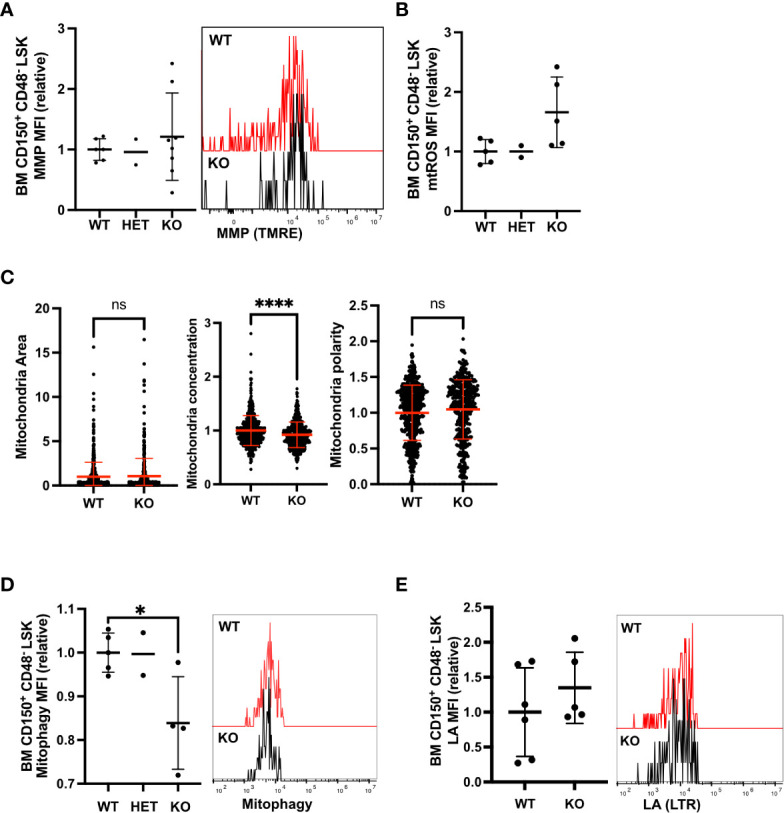
Mitophagy activity was decreased in Fancd2-deficient BM HSC. **(A)** Relative MFI (left panel) and representative flow cytometric plots (right panel) showing MMP in adult (8-12 weeks) HSCs (Fancd2 WT n=6 mice, HET n=2 mice, KO n=8 mice, left panel). **(B)** Relative MFI of mtROS in adult HSCs (Fancd2 WT n=5 mice, HET n=2 mice, KO n=5 mice). **(C)** Mitochondrial area, mitochondrial concentration and mitochondrial polarity in HSCs. HSCs were stained with anti-Tomm20 and analyzed by IFM (Fancd2 WT n=559 cells, KO n=431 cells). **(D)** Relative MFI (left panel) and representative flow cytometric plots (right panel) of mitophagy in BM HSCs (Fancd2 WT n=5 mice, HET n=2 mice, KO n=4 mice). **(E)** Relative MFI (left panel) and representative FACS plots (right panel) of LA in BM HSCs (Fancd2 WT n=6 mice, KO n=5 mice, left panel). All relative data are divided with average of controls (WT or HET) and show mean ± SD. *P<0.05, ****P<0.0001, ns, not significant.

Next, we focused on whether Fancd2 deficiency affected mitochondrial metabolism in quiescent adult BM HSCs, which do not exhibit spontaneous RS (5). While there was variation, Fancd2 KO BM HSCs exhibited comparable MMP to Fancd2 WT BM HSCs and HSPCs (**Figures 3A**, **S2A, B**). mtROS was slightly upregulated in Fancd2 KO BM HSCs but was not altered in HSPCs (**Figures 3B**, **S2C**). Mitochondria distribution was diffused and not polar in Fancd2 BM KO HSCs (**Figure 3C**). These data indicate mitochondrial activity in FANCD2-deficient BM HSCs was comparable to WT cells. We further observed that mitophagy activity significantly decreased in Fancd2 KO BM HSCs (**Figures 3D**, **S2E**), whereas lysosome activity did not change in Fancd2 KO BM HSCs (**Figures 3E**, **S2F**). Consistent with existing reports (10), these data illustrate that FANCD2-deficiency significantly decreases mitophagy in quiescent adult BM HSCs.

### FANCD2-deficiency increases metabolic activity and mitophagy in FL HSC

**Figure 4:**
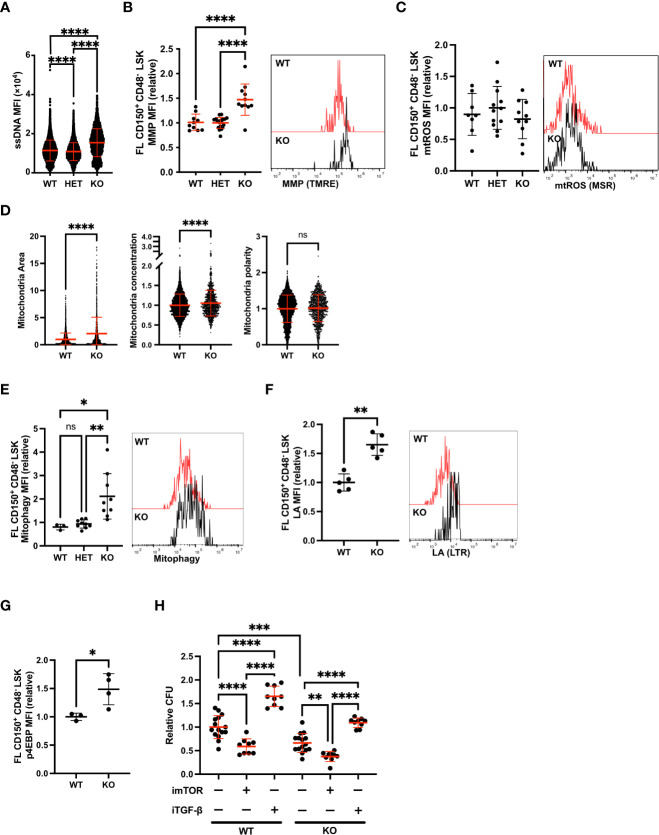
Fancd2 deficiency increases MMP and mitophagy in FL HSC. **(A)** MFI of AF-488 ssDNA in HSPCs (LSK) at E14.5 (Fancd2 WT n=7003 cells, Het n=12954 cells, KO n=2673 cells). IF of ssDNA was performed and data are analysed by IFM. **(B)** Relative MFI (left panel) and representative flow cytometric plots (right panel) of MMP in FL HSCs at E13.5 (CD150+ CD48- c-Kit+ Sca-1 Lin- (LSK)) (Fancd2 WT n=10 mice, HET n=19 mice, KO n=11 mice, left panel). **(C)** MFI (left panel) and representative flow cytometric plots (right panel) of mtROS in FL HSCs at E13.5 (Fancd2 WT n=8 mice, HET n=12 mice, KO n=10 mice, left panel). **(D)** Mitochondrial area, mitochondrial concentration and mitochondrial polarity in HSPCs (LSK) at E14.5 (Fancd2 WT n=4446 cells, KO n=987 cells). IF of Tomm20 was performed and data are analysed by IFM. **(E)** Relative MFI (left panel) and representative flow cytometric plots (right panel) of mitophagy in FL HSCs at E13.5 (Fancd2 WT n=3 mice, HET n9 mice, KO n=9 mice, left panel) **(F)** Relative MFI (left panel) and representative flow cytometric plots (right panel) of LA in FL HSCs at E13.5 (Fancd2 WT n=5 mice, HET n=4 mice, KO n=4 mice). **(G)** MFI of p4EBP in E13.5 FL HSCs (Fancd2 WT n=3 mice, KO n=4 mice). **(H)** CFU assay of HSCs with Rapamycin (imTOR) or SD-208 (iTgf-β) or untreatment (UT). Some independent experiments were performed and data are shown as relative numbers to the average of each WT UT (Fancd2 WT UT n=15 imTOR n=9 dishes, iTgf-β n=9 dishes, Fancd2 KO UT n=15 imTOR n=9 dishes, iTgf-β n=9 ishes). All relative data are divided with average of controls (WT or HET) and show mean ± SD. *P<0.05, **P<0.01, ***P<0.001, ****P<0.0001, ns, not significant.

Fetal HSCs show increased mitochondrial metabolisms compared to adult BM HSCs (9). Here, we focused on how FANCD2-deficiency changes mitochondrial metabolism in the rapidly proliferating FL HSCs. Unlike Fancd2 KO BM, FL HSCs showed an increased RS response, illustrated by characteristic gains in ssDNA (**Figure 4A**) (5). We first measured MMP and found it to be significantly higher in Fancd2 KO FL HSCs and HSPCs compared to WT and Fancd2 heterozygous (HET) (**Figures 4B**, **S3A–G**), while mtROS levels were unchanged in Fancd2 KO FL HSCs (**Figure 4C**). Further, Fancd2 KO FL HSPCs exhibited a significant increase in mitochondrial area, while the distribution of mitochondria was concentrated and polarized, indicating an overall increase in mitochondrial activity (**Figure 4D**). We also measured mitophagy and lysosome acidification in FL HSC. In contrast to adult BM HSCs, mitophagy and lysosome acidification were all upregulated in Fancd2 KO FL HSCs and HSPCs (**Figures 4E, F**, **S3H, I**).

mTOR signaling is closely associated with mitochondrial metabolism and lysosome biogenesis in HSCs (22). We thus analyzed changes in phosphorylated 4EBP (p4EBP), a direct substrate of mTOR and OPP incorporation as a measure of global protein synthesis in Fancd2 KO FL HSCs. Both measures of metabolic activity were significantly increased (**Figures 4G**, **S3J**). These data indicate that FANCD2 deficiency stimulates mitochondrial activity during fetal development in concert with an increase in mTOR signaling. Additionally, we performed CFU with treatment of mTOR inhibitor (imTOR) of Rapamycin or Tgf-β inhibitor (i Tgf-β) which rescued the RS of Fancd2 KO FL HSPCs (5). Result showed CFU number was significantly decreased in imTOR treated Fancd2-KO HSC compared to untreated Fancd2-KO HSC indicating upregulated metabolism protect from RS (**Figure 4H**). Collectively, our data indicate the RS response results increasing metabolism including mTOR pathway, mitochondrial activity and mitophagy and it sustains HSC function in FANCD2-deficient FL HSCs. These results mirror experimental observations following experimental RS in WT BM HSCs (**Figure S3K**).

## Discussion

In this study, we extended our observations on the unique fetal HSC phenotype in FA. We showed that fetal RS, in the context of FA results in a concurrent increase in mitochondrial metabolism and mitophagy. In contrast, adult quiescent BM HSCs show lower levels of mitochondrial metabolism and decreased mitophagy in Fancd2 KO. These observations provide an *in vivo* link of RS with mitochondrial metabolism and mitophagy. In 5-FU-treated mice, prominent mitochondrial changes were observed at day 6 after administration, the phase when HSCs are subjected to proliferation (15). Both FL HSCs and BM HSCs respond to RS by increasing mitochondrial metabolism and mitophagy, which suggests an inherent HSC response to RS. However, the change in mtROS and morphology in FANCD2-deficient FL HSCs and 5-FU BM HSCs differed. FL HSCs exhibit higher mtROS levels compared to BM HSC (9). mtROS level closely reflects mitochondrial quality and investigating how fetal and adult HSCs differ in mitochondrial quality control processes would be addressed in the future.

We previously described the role of RS in decreasing FA FL HSCs (5). Metformin, an inhibitor of mitochondrial complex I, was reported to ameliorate adult FANCD2-deficient HSC decline in number and potential (13) suggesting a link between FA and fetal mitochondrial metabolism. However, low placental penetration of metformin prevented its use to investigate the effect on FL HSCs (data not shown).

Previous reports showed defective mitophagy as a non-canonical phenotype of FA cells and illustrate the role of several FA proteins in mitophagy (10, 11). Our results revealed a progressive decrease in mitophagy through development. While a mechanism has not been established, the investigation should be done in the future.

The direct correlation between RS, mitochondrial metabolism and mitophagy is difficult to explain. Our data indicate that mTOR signaling and mTOR-related lysosomal biogenesis may sense RS and increase overall metabolic activity including c-Myc (23) and ribosome biogenesis (24), though further investigation is needed. In conclusion, this manuscript provides evidence for the involvement of RS in the metabolic regulation of HSCs by FANCD2 deficiency and highlights the unique characteristics of the FL HSCs compared to adult BM HSCs.

## Data availability statement

The original contributions presented in the study are included in the article/**Supplementary Material**. Further inquiries can be directed to the corresponding authors.

## Ethics statement

The animal study was reviewed and approved by TWMU animal experiment committee.

## Author contributions

MM-K and NO performed experiments. MM-K, KF, SA, PK, AN-I wrote manuscript, MG, AI, KS and AN-I funded research. All authors contributed to the article and approved the submitted version.

## Supplementary material

Supplementary Figure 1 APH-treated HSC of phenotypes in ex vivo. **(A-C)** Representative flow cytometric plots **(A)** and relative frequency to total live cells **(B)** and total analyzed cell count **(C)** of CD150+ CD48- c-Kit+ Lin- HSC in d0, d6, d12 after 5-FU injection (d0 n=3 mice, d6 n=3 mice, d12 n=3 mice). **(D, E)** Relative MFI of cell trace violet (CTV, C34557 Thermo Fisher) **(D)** and relative percentage of Annexin-V single positive (SP) cells **(E)** in BM HSCs (CD150+ c-Kit+ Lin-) ex vivo cultured for 7 days with (APH) or without APH (UT) (UT n=6 wells, APH n=6 wells). **(F)** CFU assay of BM HSCs with or without APH. 3 independent experiments were performed and data are shown as relative numbers to the average of each UT (UT n=9 dishes, APH n=9 dishes). All data show mean ± SD.

Supplementary Figure 2 Metabolism data for Fancd2-deficient BM HSPCs. **(A, B)** Relative MFI of MMP in BM HSPCs **(A)** and BM total live cells **(B)** (Fancd2 WT n=5 mice, HET n=3 mice, KO n=8 mice). **(C-E)** Relative MFI of mtROS in HSPCs **(C)** (Fancd2 WT n=5 mice, HET n=2 mice, KO n=5 mice) and relative MFI of mitophagy **(D)** (Fancd2 WT n=5 mice, HET n=2 mice, KO n=5 mice), MFI of lysosome acidification **(E)** (Fancd2 WT n=6 mice, KO n=5 mice). All data show mean ± SD.

Supplementary Figure 3 Metabolism data for Fancd2-deficient FL HSPCs. **(A)** Scheme for FA deficient FL HSC. **(B-D)** Representative flow cytometric plots **(B)** and relative frequency to total live cells **(C)** and total analyzed cell count **(D)** of CD150+ CD48- c-Kit+ Sca-1+ Lin- WT and Fancd2 KO E13.5 FL HSC. **(E)** Relative frequency of HSPCs to total live cell. **(F, G)** Relative MFI of MMP in E13.5 FL HSPCs **(F)** and total live cells (G, A-G, Fancd2 WT n=10 mice, HET n=19 mice, KO n=11 mice). **(H, I)** Relative MFI of mitophagy (H, Fancd2 WT n=3 mice, HET n=8 mice, KO n=9 mice, BM HSC n=3 mice: mom of pups, Fancd2 HET) and MFI of LA **(I)** (Fancd2 WT n=5 mice, HET n=4 mice, KO n=5 mice) in E13.5 FL HSCs and BM HSCs. **(J)** MFI of OPP in E13.5 FL HSCs and BM HSCs (Fancd2 WT n=5 mice, HET n=6 mice, KO n=5 mice, BM HSC n=2 mice: mom of pups, Fancd2 HET). **(K)** Scheme for RS increases mitochondrial metabolism and mitophagy in FANCD2 deficient FL HSC. How mTOR signal senses RS should be described in the future. All data show mean ± SD. *P<0.05, **P<0.01, ****P<0.0001.
