# Supplementary figures and images for: Replication stress increases mitochondrial metabolism and mitophagy in FANCD2 deficient fetal liver hematopoietic stem cells
Source: Front Oncol. 2023 Mar 7;13:1108430. doi: 10.3389/fonc.2023.1108430 (PMC10061350; doi:10.3389/fonc.2023.1108430)

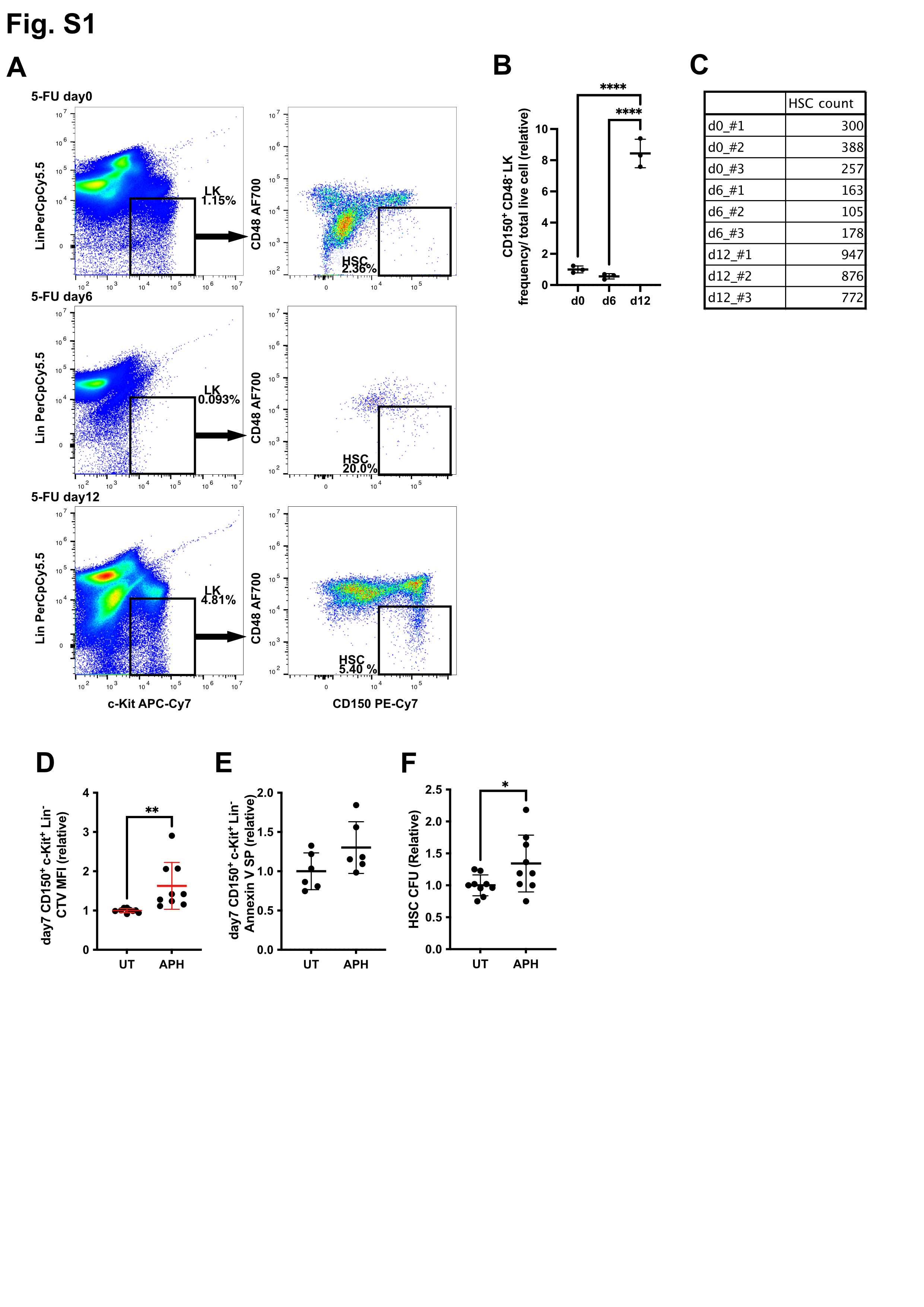

Supplement: Supplementary Figure 1 — APH-treated HSC of phenotypes in ex vivo. (A-C) Representative flow cytometric plots (A) and relative frequency to total live cells (B) and total analyzed cell count (C) of CD150+ CD48- c-Kit+ Lin- HSC in d0, d6, d12 after 5-FU injection (d0 n=3 mice, d6 n=3 mice, d12 n=3 mice). (D, E) Relative MFI of cell trace violet (CTV, C34557 Thermo Fisher) (D) and relative percentage of Annexin-V single positive (SP) cells (E) in BM HSCs (CD150+ c-Kit+ Lin-) ex vivo cultured for 7 days with (APH) or without APH (UT) (UT n=6 wells, APH n=6 wells). (F) CFU assay of BM HSCs with or without APH. 3 independent experiments were performed and data are shown as relative numbers to the average of each UT (UT n=9 dishes, APH n=9 dishes). All data show mean ± SD. [file Image_1.tiff]

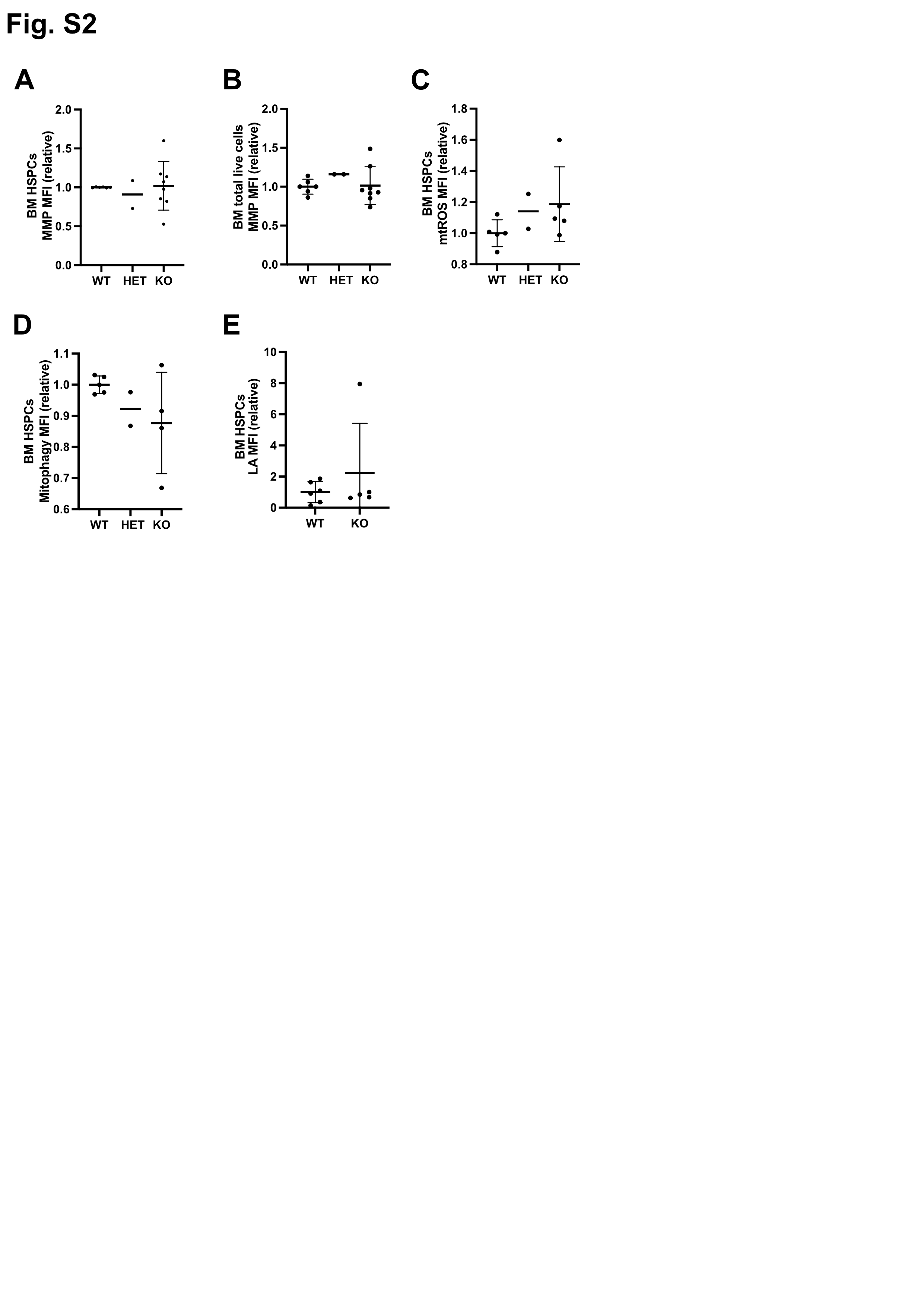

Supplement: Supplementary Figure 2 — Metabolism data for Fancd2-deficient BM HSPCs. (A, B) Relative MFI of MMP in BM HSPCs (A) and BM total live cells (B) (Fancd2 WT n=5 mice, HET n=3 mice, KO n=8 mice). (C-E) Relative MFI of mtROS in HSPCs (C) (Fancd2 WT n=5 mice, HET n=2 mice, KO n=5 mice) and relative MFI of mitophagy (D) (Fancd2 WT n=5 mice, HET n=2 mice, KO n=5 mice), MFI of lysosome acidification (E) (Fancd2 WT n=6 mice, KO n=5 mice). All data show mean ± SD. [file Image_2.tiff]

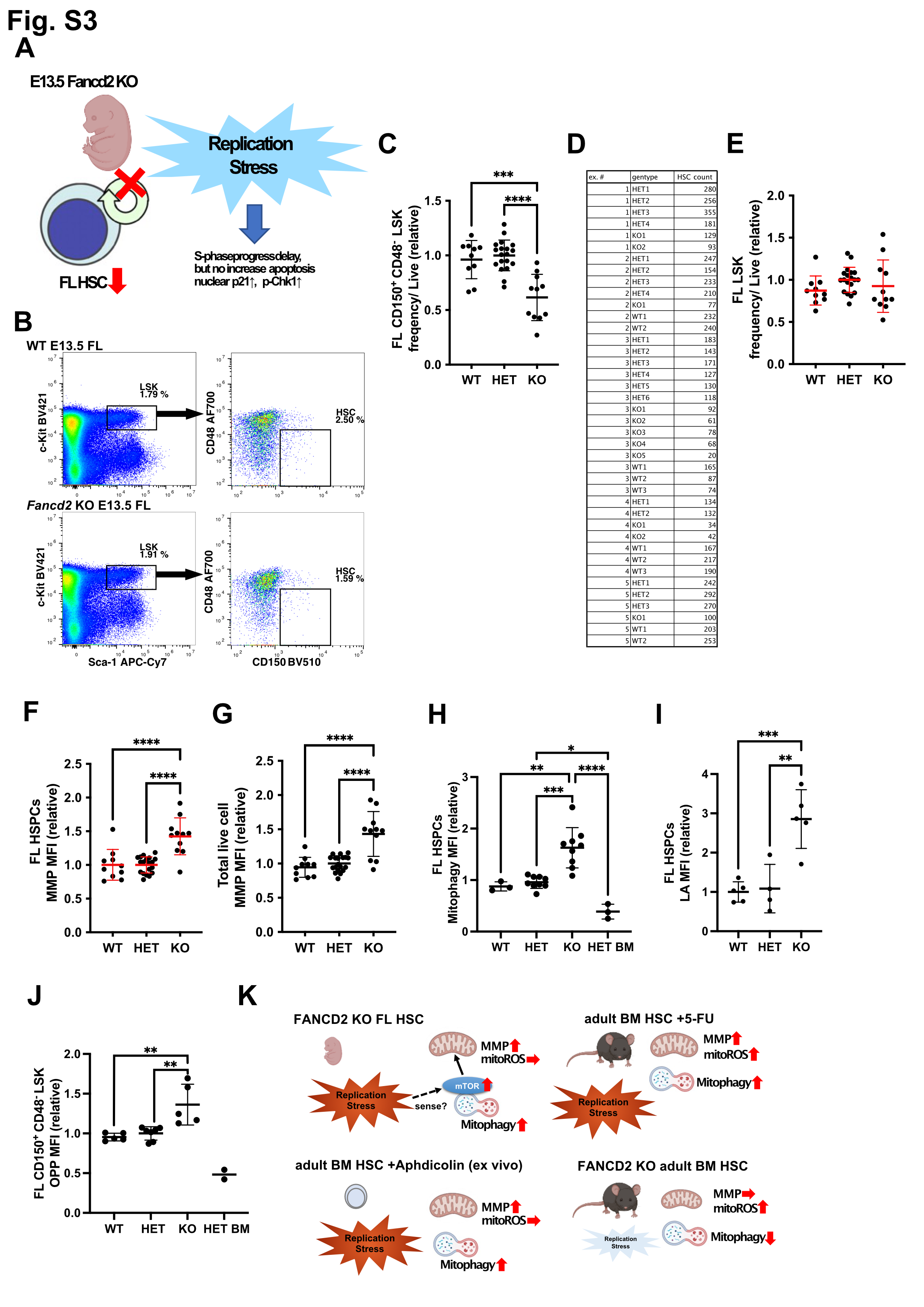

Supplement: Supplementary Figure 3 — Metabolism data for Fancd2-deficient FL HSPCs. (A) Scheme for FA deficient FL HSC. (B-D) Representative flow cytometric plots (B) and relative frequency to total live cells (C) and total analyzed cell count (D) of CD150+ CD48- c-Kit+ Sca-1+ Lin- WT and Fancd2 KO E13.5 FL HSC. (E) Relative frequency of HSPCs to total live cell. (F, G) Relative MFI of MMP in E13.5 FL HSPCs (F) and total live cells (G, A-G, Fancd2 WT n=10 mice, HET n=19 mice, KO n=11 mice). (H, I) Relative MFI of mitophagy (H, Fancd2 WT n=3 mice, HET n=8 mice, KO n=9 mice, BM HSC n=3 mice: mom of pups, Fancd2 HET) and MFI of LA (I) (Fancd2 WT n=5 mice, HET n=4 mice, KO n=5 mice) in E13.5 FL HSCs and BM HSCs. (J) MFI of OPP in E13.5 FL HSCs and BM HSCs (Fancd2 WT n=5 mice, HET n=6 mice, KO n=5 mice, BM HSC n=2 mice: mom of pups, Fancd2 HET). (K) Scheme for RS increases mitochondrial metabolism and mitophagy in FANCD2 deficient FL HSC. How mTOR signal senses RS should be described in the future. All data show mean ± SD. *P<0.05, **P<0.01, ****P<0.0001. [file Image_3.tiff]
